# Intermittent versus continuous renal replacement therapy in acute methanol poisoning: comparison of clinical effectiveness in mass poisoning outbreaks

**DOI:** 10.1186/s13613-017-0300-7

**Published:** 2017-07-20

**Authors:** Sergey Zakharov, Jan Rulisek, Olga Nurieva, Katerina Kotikova, Tomas Navratil, Martin Komarc, Daniela Pelclova, Knut Erik Hovda

**Affiliations:** 1 Department of Occupational Medicine, Toxicological Information Center, First Faculty of Medicine, Charles University and General University Hospital, Prague, Czech Republic; 2 Department of Anesthesia and Intensive Care, First Faculty of Medicine, Charles University and General University Hospital, Prague, Czech Republic; 3 Department of Biomimetic Electrochemistry, J. Heyrovský Institute of Physical Chemistry of CAS, v.v.i, Prague, Czech Republic; 4 Institute of Biophysics and Informatics, First Faculty of Medicine, Charles University and General University Hospital, Prague, Czech Republic; 5 The Norwegian CBRNE Centre of Medicine, Department of Acute Medicine, Oslo University Hospital, Oslo, Norway

**Keywords:** Methanol poisoning, Mass poisoning outbreak, Continuous renal replacement therapy, Intermittent hemodialysis, Treatment outcome, Effectiveness

## Abstract

**Background:**

Intermittent hemodialysis (IHD) is the modality of choice in the extracorporeal treatment (ECTR) of acute methanol poisoning. However, the comparative clinical effectiveness of intermittent versus continuous modalities (CRRT) is unknown. During an outbreak of mass methanol poisoning, we therefore studied the effect of IHD versus CRRT on mortality and the prevalence of visual/central nervous system (CNS) sequelae in survivors.

**Methods:**

The study was designed as prospective observational cohort study. Patients hospitalized with a diagnosis of acute methanol poisoning were identified for the study. Exploratory factor analysis and multivariate logistic regression were applied to determine the effect of ECTR modality on the outcome.

**Results:**

Data were obtained from 41 patients treated with IHD and 40 patients with CRRT. The follow-up time in survivors was two years. Both groups of patients were comparable by age, time to presentation, laboratory data, clinical features, and other treatment applied. The CRRT group was more acidemic (arterial blood pH 6.96 ± 0.08 vs. 7.17 ± 0.07; *p* < 0.001) and more severely poisoned (25/40 vs. 9/41 patients with Glasgow Coma Scale (GCS) ≤ 8; *p* < 0.001). The median intensive care unit length of stay (4 (range 1–16) days vs. 4 (1–22) days; *p* = 0.703) and the number of patients with complications during the treatment (11/41 vs. 13/40 patients; *p* = 0.576) did not differ between the groups. The mortality was higher in the CRRT group (15/40 vs. 5/41; *p* = 0.008). The number of survivors without sequelae of poisoning was higher in the IHD group (23/41 vs. 10/40; *p* = 0.004). There was a significant association of ECTR modality with both mortality and the number of survivors with visual and CNS sequelae of poisoning, but this association was not present after adjustment for arterial blood pH and GCS on admission (all *p* > 0.05).

**Conclusions:**

In spite of the faster correction of the acidosis and the quicker removal of the toxic metabolite in intermittent dialysis, we did not find significant differences in the treatment outcomes between the two groups after adjusting for the degree of acidemia and the severity of poisoning on admission. These findings support the strategy of “use what you have” in situations with large outbreaks and limited dialysis capacity.

## Background

Acute methanol poisonings occur frequently either in clusters or as mass “epidemics,” representing a challenge for healthcare providers throughout the world [1–4]. Treatment consists of a buffer to correct acidemia, antidote (ethanol or fomepizole) to block the metabolism of methanol, folate substitution to enhance the endogenous metabolism of formate, and dialysis to eliminate methanol and its toxic metabolite [5–7]. Formic acid/formate anions have a strong cytotoxic effect through inhibition of the mitochondrial respiration [8, 9]. The accumulation of formic acid results in metabolic acidosis with lactacidosis, optic nerve impairment, and damage of basal ganglia, especially when its concentration rises above 10–12 mmol/L or 460–550 mg/L [10–15]. The mortality of methanol poisonings is high; severe metabolic acidosis (pH < 7.0), lack of respiratory compensation, and coma (Glasgow Coma Scale (GCS) < 8) on admission are known risk factors predicting poor outcome [1, 16–19].

The role of enhanced elimination in the treatment of acute methanol poisoning is well established. Intermittent (IHD) or extended daily hemodialysis (EDD) and continuous veno-venous hemofiltration, hemodialysis, or hemodiafiltration (CRRT) are all commonly used [20–22]. There are various reports providing data on the superiority of IHD regarding the rate of elimination of both methanol and formate [23–25], as well as correction of the acidemia [26]. Recent recommendations from the EXTRIP expert group support intermittent hemodialysis as the modality of choice in methanol poisoning, and continuous modalities as an acceptable alternative in cases of unavailability of intermittent hemodialysis [27]. However, no studies evaluating clinical endpoints comparing the short- and long-term outcomes of treatment exist.

During the mass methanol poisoning outbreak that occurred in the Czech Republic in 2012–2015, both intermittent and continuous modalities of enhanced elimination were applied [28]. We compared clinical endpoints, mortality, and the prevalence of long-term visual and central nervous system (CNS) sequelae in the patients treated with intermittent *versus* continuous modalities of hemodialysis.

## Methods

### Patients and procedures

The study was designed as a prospective observational cohort study. A detailed history of the poisoning and of the onset and dynamics of ocular and systemic toxicity was obtained in a prospective manner directly from the patients or from relatives of critically ill patients upon admission to hospital. The discharge reports of all hospitalized patients with a confirmed diagnosis of acute methanol poisoning and the results of neurological and ophthalmological examinations on admission, during hospitalization, and on discharge were collected and analyzed in the Czech Toxicological Information Center (TIC). The patients who died outside hospital and the patients treated without enhanced elimination methods were excluded from the study.

Laboratory analyses were performed on admission. Diagnosis was established when (1) a history of recent ingestion of illicit spirits was available and serum methanol was higher than 6.2 mmol/L (200 mg/L) and/or an osmolal gap (OG) ≥ 20 mOsm/kgH_2_O (that could not be explained by ethanol) was found or (2) there was a history/clinical suspicion of methanol poisoning, and serum methanol was above the limit of detection with at least two of the following: pH < 7.3, bicarbonate <20 mmol/L, and anion gap (AG) ≥ 20 mmol/L.

The clinical examination protocol included complete ocular examination with standard ophthalmologic tests, cerebral computed tomography (CT) or magnetic resonance imaging (MRI) of the brain, and standard neurological examination. The follow-up examination protocol included additionally optical coherence tomography (OCT) with retinal nerve fibers layer evaluation and visual evoked potentials (VEP). The patients were considered to have visual sequelae of acute methanol poisoning if the symptoms of toxic neuropathy of the optic nerve were documented on admission/during hospitalization, with pathologic findings on visual acuity, visual fields, color vision, contrast sensitivity, and persisting lesions on fundoscopy with other symptoms of visual damage being found on discharge from hospital [29, 30]. The patients were considered as having CNS sequelae of poisoning if symmetrical necrosis and hemorrhages of basal ganglia were present on CT or MRI of the brain [31, 32].

### Treatment

All patients were treated in accordance with the American Association of Clinical Toxicology and European Association of Poisons Centres and Clinical Toxicologists (AACT/EAPCCT) practice guidelines on the treatment of methanol poisoning [5]. Bicarbonate 8.4 or 4.2% solution was given intravenously as a buffer to the patients with metabolic acidosis. Fomepizole was given as a bolus dose of 15 mg/kg i.v. diluted in isotonic saline, then 10 mg/kg every 12 h in the patients without hemodialysis, and every 4 h during hemodialysis. From the fifth dose and on, 15 mg/kg was given in order to compensate for increased metabolism [33]. Ethanol was administered intravenously as 10% solution in 5% glucose according to the following scheme: the loading dose of approximately 800 mg/kg (7.5–8.0 ml/kg) during 1 h, followed by the maintenance dose 1.0–2.0 ml/kg/h or 2.5–3.0 ml/kg/h during the hemodialysis. If ethanol was administered per os, 0.7–1.0 ml/kg/h of 20% solution was generally applied in boluses each 3 h [34, 35]. Folates were administered to substitute the endogenous pool of folate.

Enhanced elimination was performed if the patients met any of the following criteria: serum methanol higher than 15.6 mmol/L (500 mg/L), metabolic acidosis with arterial blood pH < 7.30, or had the signs of visual toxicity [5]. The choice of modality of enhanced elimination was based on several factors, such as the hemodynamic stability of a patient on admission, or the severity of poisoning, and availability of dialysis equipment. Serum ethanol and methanol concentrations have been monitored during ECTR in all patients, and serum formate levels were measured in most of them. The initial duration of ECTR was determined based on the admission laboratory data (serum methanol, formate, arterial blood pH) and corrected based on laboratory concentration monitoring data. The IHD and CRRT prescriptions applied during mass poisoning outbreak have been presented in our previous publications [25, 26].

### Laboratory investigations

Methanol was measured using gas chromatography with flame ionization detection and a direct injection with internal standard (Gas Chromatograph Chrom 5, Laboratory Instruments Prague, Czech Republic), limit of detection 1.9 mmol/L (60 mg/L), and day-to-day coefficient of variation 2.5–5.4%. Formate was measured enzymatically on a Hitachi analyzer (Hitachi 912, Hitachi Science Systems Ltd., Japan) using formate dehydrogenase (Roche, France) and nicotinamide adenine dinucleotide (NAD) (Roche, France). Serum ethanol was analyzed by gas chromatography with flame ionization detection and a direct injection with internal standard (Gas Chromatograph Chrom 5, Laboratory Instruments Prague, Czech Republic). Limit of detection was 0.9 mmol/L (40 mg/L) and day-to-day coefficient of variation 3.8–7.1%.

### Calculations and data analysis

The data were expressed as means with confidence interval (significance level *α* = 0.05) or summarized as absolute frequencies and percentages where appropriate. When comparing the groups of patients treated with different modalities of ECTR, the independent-groups *t* test (normally distributed variables), Mann–Whitney *U* test (non-normally distributed variables), or Chi-square test (frequency counts) was used. All the patients hospitalized with acute methanol poisoning and treated with hemodialysis have been included without analysis of outliers due to the limited size of the study population.

The univariate logistic regression predicting death and survival with visual/CNS sequelae was performed and further adjusted for possible confounders. The collinearity of the variables was present and assessed by Spearman’s rank correlation analysis. For this reason, exploratory factor analysis (EFA, principal component analysis) was performed since a relatively high number of moderately to strongly correlated confounders was identified by Spearman’s rank correlation. EFA identified arterial blood pH, serum creatinine, glucose, ethanol, GCS on admission, and dialysis modality as factors for inclusion into the logistic regression model. For each dependent parameter, the univariate and consequent multivariate logistic regression analysis was performed. The best subset variable selection process was applied to develop the final model. To evaluate goodness of fit of the logistic regression models, Hosmer–Lemeshow pseudo-*R* ^2^ and Hosmer–Lemeshow Chi-square test were performed.

All statistical calculations including logistic regression analyses were carried out with a level of significance *α* = 0.05. Statistical analysis was performed using Excel (Microsoft, USA), and the formal calculations were produced in QC Expert software 3.1 (Trilobyte, Pardubice, Czech Republic) and in IBM SPSS version 23.0 and Statistica SW version 10.0.

## Results

**Fig. 1:**
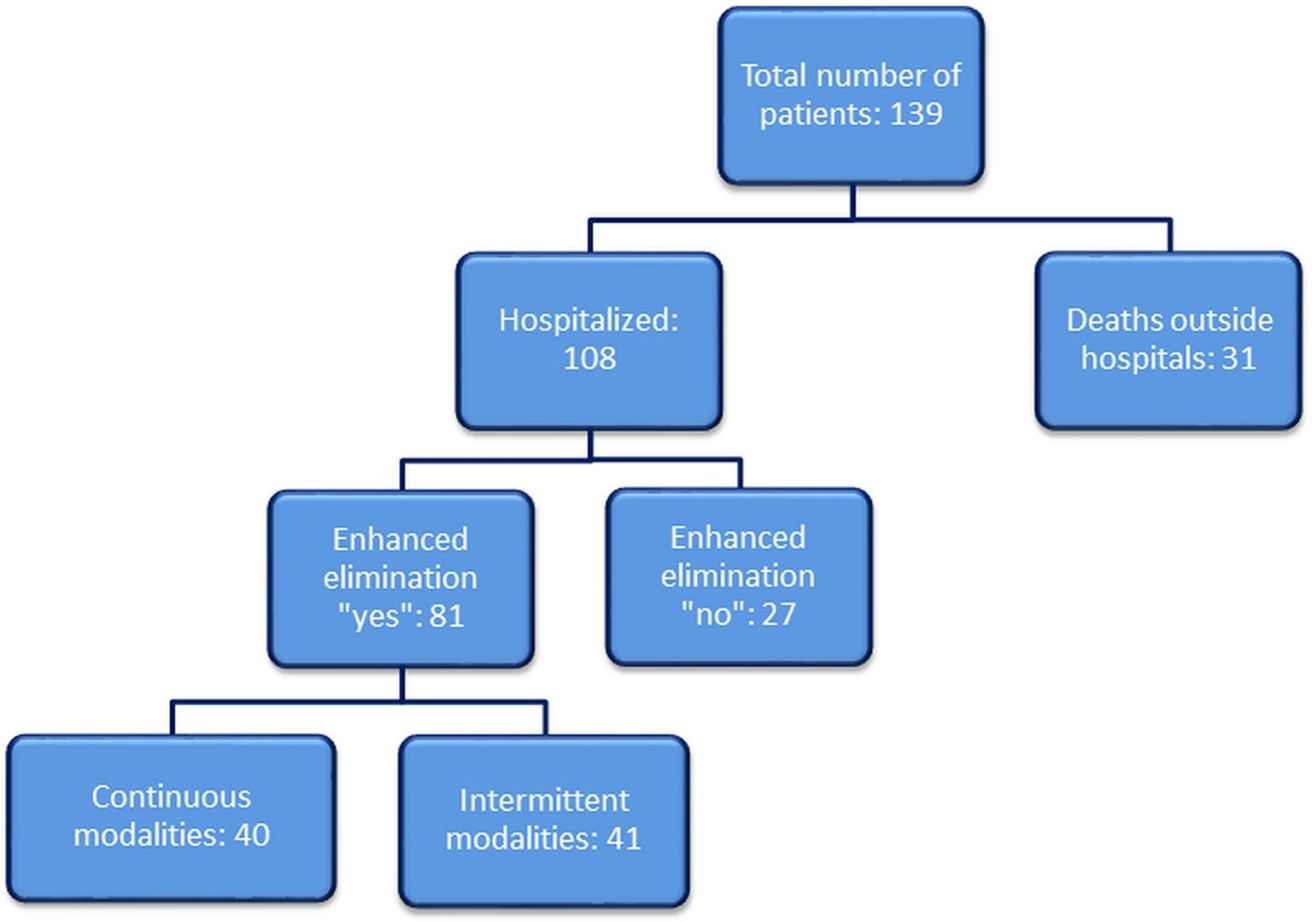
Flowchart of the patients in the study

A total of 139 cases of methanol poisoning occurred during the period from the September 3, 2012, until the December 31, 2015, of whom 108 patients were treated in hospital (Fig. 1). Among the 108 hospitalized patients, extracorporeal treatment (ECTR) methods were applied in 81 patients (IHD in 36 patients, EDD in 5 patients, and CRRT in 40 patients). Taking into account the small number of patients treated with EDD, we combined the patients treated with EDD and IHD in one group for further analysis, given the closer resemblance between EDD and IHD, compared to EDD and CRRT. Excluding the EDD from the analysis did not change the results.

The laboratory data and the clinical features on admission are presented in Tables 1 and 2. Regarding the time of presentation, 9% were admitted within 12 h of the methanol ingestion, 21% within 13–24 h, 38% within 25–48 h, and 15% later than 48 h. In 17% of the cases, it was impossible to identify the time between the consumption of toxic alcohol and admission to hospital. The type of alcohol was known in 78 patients and the approximate quantity in 65 cases. All samples of toxic alcohol contained mixtures of methanol and ethanol, but the final proportion varied substantially, from 20% methanol/80% ethanol to 50% methanol/50% ethanol, in different kinds of strong alcoholic beverages with an alcohol content of around 40% ABV (alcohol by volume, or v/v).

**Table 1:** Baseline characteristics and laboratory data on admission in the groups of patients treated with different dialysis modalities, IHD versus CRRT (*n* = 81; means with 95%CI)

Group of patients	Age, years	S-MetOH, mmol/L (g/L)	S-EtOH, mmol/L (g/L)	S-Formate, mmol/L (g/L)	S-Lactate, mmol/L (g/L)	Arterial blood pH	pCO_2_, kPa	HCO_3_^−^, mmol/L (g/L)	BD, mmol/L	Creatinine, µmol/L (mg/L)	Glucose, mmol/L (g/L)	Time to presentation, hours
IHD (*n* = 41; 9F)	50.4 ± 4.2	40.9 ± 10.9 1.31 ± 0.35	9.8 ± 6.3 0.45 ± 0.29	13.3 ± 3.0 0.59 ± 0.14	4.4 ± 1.6 0.39 ± 0.14	7.17 ± 0.07	4.5 ± 1.8	11.3 ± 2.2 (0.67 ± 0.13)	−16.5 ± 3.2	93.3 ± 9.5 (10.5 ± 1.1)	8.5 ± 1.2 (1.53 ± 0.22)	41 ± 7
CRRT (*n* = 40; 8F)	51.5 ± 4.2	58.3 ± 16.5 1.87 ± 0.53	4.8 ± 5.2 0.22 ± 0.24	15.9 ± 3.0 0.68 ± 0.14	7.1 ± 1.7 0.63 ± 0.15	6.96 ± 0.08	4.1 ± 0.7	8.0 ± 2.0 (0.49 ± 0.12)	−21.7 ± 4.2	110.0 ± 13.0 (12.44 ± 0.34)	11.2 ± 1.8 (1.98 ± 0.32)	36 ± 6
Total (*n* = 81; 17F)	50.9 ± 2.9	49.6 ± 10.0 (1.59 ± 0.32)	7.2 ± 4.1 (0.33 ± 0.19)	14.5 ± 2.1 (0.63 ± 0.09)	5.9 ± 1.2 (0.53 ± 0.11)	7.06 ± 0.06	4.3 ± 0.9	9.6 ± 1.5 (0.59 ± 0.09)	−19.1 ± 2.6	101.7 ± 8.1 (11.43 ± 0.92)	9.8 ± 1.1 (1.77 ± 0.20)	39 ± 5
P _IHD/CRRT_	0.724	0.079	0.230	0.208	*0.024*	*<0.001*	0.609	*0.024*	*0.048*	*0.042*	*0.012*	0.274

Italic text indicates statistically significant result at *p* < 0.05

*IHD* intermittent hemodialysis,* CRRT* continuous renal replacement therapy,* F* females,* S* serum,* BD* base deficit,* MetOH* methanol, *EtOH* ethanol

**Table 2:** Clinical parameters on admission in the groups of patients treated with different dialysis modalities, IHD versus CRRT (*n* = 81; means with 95%CI)

Group of patients	MAP, mmHg (kPa)	GCS	Coma, *n* (%)	RR, min	MV, *n* (%)	HR, min	Vasopressors/inotropes	VD, *n* (%)	D, *n* (%)	GI, *n* (%)	CA and RA, *n* (%)	Alcoholism, *n* (%)
IHD (*n* = 41; 9F)	106.1 ± 6.0 (14.15 ± 0.80)	13 ± 2	8 (20%)	19 ± 1	9 (22%)	87 ± 5	6 (15%)	17 (41%)	11 (27%)	21 (51%)	2 (5%)	19 (46%)
CRRT (*n* = 40; 8F)	96.0 ± 7.1 (12.80 ± 0.95)	8 ± 2	25 (63%)	20 ± 2	28 (70%)	96 ± 7	23 (58%)	22 (55%)	22 (55%)	23 (58%)	5 (13%)	20 (50%)
Total (*n* = 81; 17F)	101.1 ± 4.9 (13.48 ± 0.65)	10 ± 1	33 (41%)	19 ± 1	37 (46%)	92 ± 4	29 (36%)	39 (48%)	33 (41%)	44 (54%)	7 (9%)	39 (48%)
P _IHD/CRRT_	*0.026*	*<0.001*	*<0.001*	0.257	*<0.001*	*0.041*	*<0.001*	0.223	*0.010*	0.570	0.222	0.742

Italic text indicates statistically significant result at *p* < 0.05

*IHD* intermittent hemodialysis,* CRRT* continuous renal replacement therapy, coma = GCS < 8,* GCS* Glasgow Coma Scale,* VD* visual disturbances,* D* dyspnea,* GI* gastrointestinal symptoms,* HR* heart rate,* CA* cardiac arrest,* RA* respiratory arrest,* RR* respiratory rate,* MAP* mean arterial pressure,* MV* mechanical ventilation

Treatment given to the patients, the median intensive care unit length of stay (ICU LOS), and the outcome are presented in Table 3. In the IHD group, two cases of filter clotting, two cases of severe hypotension, and one episode of rebound of metabolic acidosis after termination of dialysis due to set clotting occurred. The most common complications during the treatment were delirium tremens (*n* = 3) and bleeding due to thrombocytopenia or heparinization (*n* = 3). Other complications included pulmonary embolism, pneumonia, sepsis, and thrombophlebitis (all *n* = 1).

**Table 3:** Treatment provided and outcome in the groups of patients treated with different dialysis modalities, IHD versus CRRT (*n* = 81)

	Treatment given								Complications and outcome		
Group of patients	Ethanol, *n* (%)	Fomepizole, *n* (%)	Alkalinization, *n* (%)	HD start, hours	HD duration, hours	Folate therapy, *n* (%)	Complications, *n* (%)	ICU LOS, days	Survivors without sequelae	Survived with sequelae	Died, *n* (%)
IHD (*n* = 41; 9F)	28 (68%)	13 (32%)	24 (59%)	2.5 ± 0.6	9.1 ± 2.4	31 (76%)	11 (27%)	4 (1–16)	23 (56%)	13 (32%)	5 (12%)
CRRT (*n* = 40; 8F)	27 (68%)	12 (30%)	33 (83%)	3.5 ± 0.9	45.7 ± 9.7	34 (86%)	13 (33%)	4 (1–22)	10 (25%)	15 (37.5%)	15 (37.5%)
Total (*n* = 81)	55 (68%)	25 (31%)	57 (70%)	3.0 ± 0.6	25.8 ± 6.3	65 (80%)	25 (31%)	4 (1–22)	33 (40%)	28 (35%)	20 (25%)
P _IHD/CRRT_	0.939	0.868	*0.018*	0.084	*<0.001*	0.289	0.576	0.703	*0.004*	0.584	*0.008*

Italic text indicates statistically significant result at *p* < 0.05

*IHD* intermittent hemodialysis,* CRRT* continuous renal replacement therapy, HD start—time from hospital admission to ECTR initiation, HD duration—the total duration of RRT sessions (incorporates both single and multiple sessions of IHD), ICU LOS—ICU length of stay (median with range)

Among the patients treated with CRRT, one episode of rebound of metabolic acidosis after termination of CVVHD due to filter clotting occurred. Other complications seen during the treatment were pneumonia (*n* = 8), delirium tremens (*n* = 6), sepsis (*n* = 4), and bleeding due to heparinization (*n* = 2). All the patients with delirium tremens had a history of chronic alcohol abuse and the complication developed after discontinuation of ethanol administration. Finally, elevation of amylase enzyme, paresis of abducens nerve, sepsis, and anasarca were also seen (all *n* = 1).

**Fig. 2:**
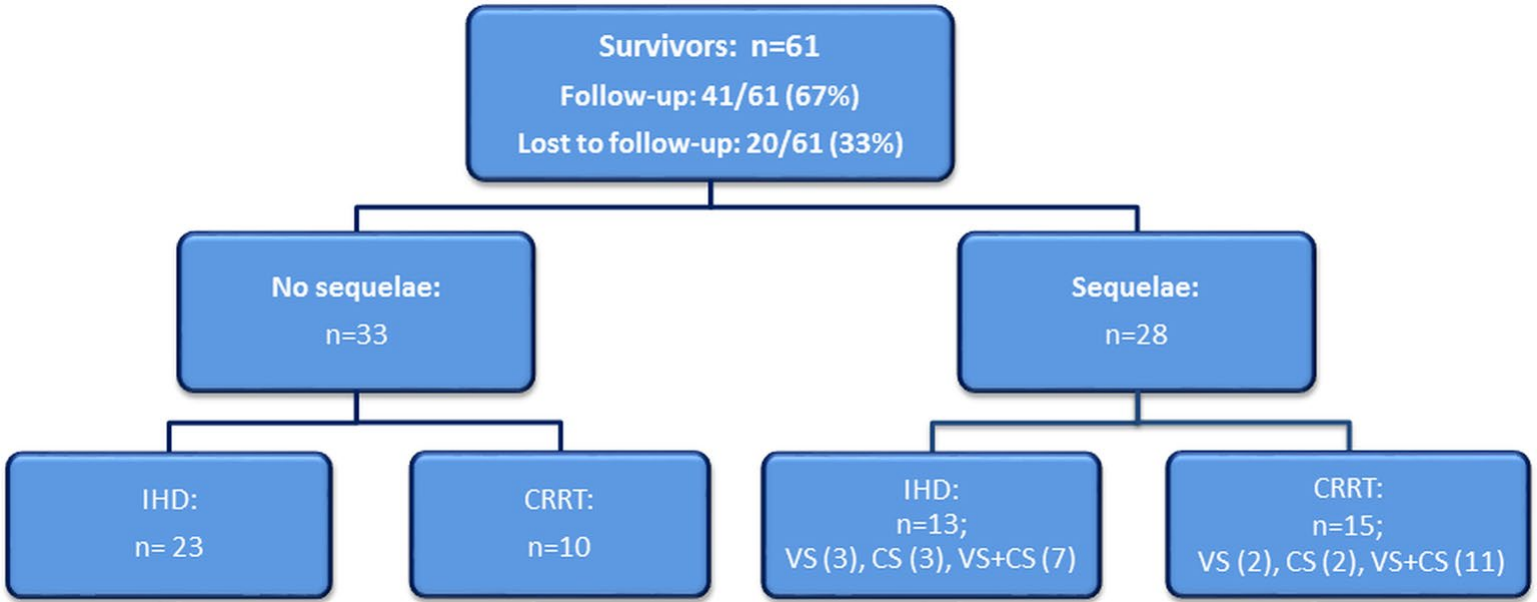
Flowchart of the treatment outcome in the survivors of poisoning in the study (VS—visual sequelae, CS—central nervous system sequelae)

A clinical follow-up examination was conducted in the survivors of poisoning three to eight months and two years after discharge from hospital (Fig. 2). In the survivors lost to follow-up, the information on the outcome and sequelae of poisoning was extracted from the discharge reports. Visual sequelae (VS), central nervous system sequelae (CS), or both VS and CS sequelae were present in 46% of survivors treated with ECTR.

A significant association was found between the mortality rate and the mode of enhanced elimination (*p* = 0.008). Concerning the prevalence of visual and CNS sequelae of poisoning, an association was found with arterial blood pH, creatinine, glucose (all *p* < 0.001), and the mode of enhanced elimination (<0.001). Further, significant association was present between the prevalence of sequelae and GCS (*p* < 0.001). No association was found between the treatment outcome and age, sex, serum methanol, formate, time to presentation and time to start of hemodialysis, type of antidote administered, and folate therapy (all *p* > 0.05).

The results of univariate and multivariate logistic regression analysis of impact of different parameters including hemodialysis modality on the treatment outcome (death or survival with visual and/or CNS sequelae of poisoning) are presented in Tables 4 and 5. After adjustment for arterial blood pH on admission, the impact of ECTR modality on both mortality and survival with health sequelae of poisoning was not significant. The same result was received after adjustment for GCS on admission (all *p* > 0.05).

**Table 4:** Univariate logistic regression analysis of impact of different parameters including hemodialysis modality (IHD vs. CRRT) on mortality and survival with sequelae in the patients with acute methanol poisoning (*n* = 81)

Variable	Outcome							
	Mortality				Survival with long-term visual/CNS sequelae			
	OR	(95% CI)	*p*	*R* ^2^	OR	(95% CI)	*p*	*R* ^2^
HD modality (IHD vs. CRRT)	*0.231*	*0.075–0.719*	*0.011*	*0.127*	*0.261*	*0.101–0.671*	*0.005*	*0.131*
Arterial blood pH	*0.002*	*0.000–0.038*	*<0.001*	*0.419*	*0.000*	*0.000–0.010*	*<0.001*	*0.546*
GCS	*0.756*	*0.663–0.862*	*<0.001*	*0.412*	*0.768*	*0.679–0.868*	*<0.001*	*0.384*
S-creatinine	*1.027*	*1.011–1.043*	*0.001*	*0.229*	*1.041*	*1.019–1.064*	*<0.001*	*0.323*
S-glucose	*1.179*	*1.059–1.312*	*0.003*	*0.174*	*1.164*	*1.034–1.310*	*0.012*	*0.128*
S-EtOH	0.948	0.813–1.106	0.498	0.331	*0.999*	*0.998–1.000*	*0.042*	*0.123*

The alpha level used in the univariate analysis is *α* = 0.05

Italic values indicate statistically significant result at *p* < 0.05

*OR* odds ratio,* CI* confidence interval, HD modality—hemodialysis modality, arterial blood pH—arterial blood pH on admission,* GCS* Glasgow Coma Scale on admission,* S* serum,* EtOH* ethanol

**Table 5:** Mulivariate logistic regression analysis of hemodialysis modality impact on outcomes adjusted for a) arterial blood pH and b) GCS in the patients with acute methanol poisoning (*n* = 81)

Variable	Outcome							
	Mortality				Survival with long-term visual/CNS sequelae			
	OR	(95% CI)	*p*	*R* ^2^	OR	(95% CI)	*p*	*R* ^2^
a)				*0.427*				*0.551*
HD modality (IHD vs. CRRT)	0.589	0.150–2.318	0.449		0.637	0.181–2.239	0.482	
Arterial blood pH	*0.003*	*0.000–0.062*	*<0.001*		*0.000*	*0.000–0.015*	*<0.001*	
b)				*0.400*				*0.391*
HD modality (IHD vs. CRRT)	0.585	0.151–2.265	0.438		0.632	0.201–1.988	0.433	
GCS	*0.786*	*0.669–0.880*	*<0.001*		*0.783*	*0.687–0.891*	*<0.001*	

The alpha level used in the univariate analysis is *α* = 0.05

Italic values indicate statistically significant result at *p* < 0.05

*OR* odds ratio,* CI* confidence interval, HD modality—hemodialysis modality, arterial blood pH—arterial blood pH on admission,* GCS* Glasgow Coma Scale on admission,* S* serum,* EtOH* ethanol

## Discussion

In spite of the faster correction of the acidosis [26] and the quicker removal of the toxic metabolite in IHD as compared to CRRT [25], we did not find significant differences in the treatment outcomes between the two groups after adjusting for the degree of acidemia and the severity of poisoning on admission. We found no difference in clinical effectiveness between the two modalities of ECTR regarding mortality rate, rate of survival with long-term visual and/or CNS sequelae. Likewise, there are no clinical data supporting the superiority in the existing literature, despite the consensus recommending IHD as the modality of choice in acute methanol poisoning [27].

There is a variety of reasons why randomized clinical trials (RCT) comparing the effectiveness of different modalities of enhances elimination are difficult to perform, including infrequent poisonings, often lack of availability of dialysis equipment where the large outbreaks occur, obvious ethical concerns, and so on [4]. Also, most of the outbreaks are limited in size and time, making the planning of a RCT a challenging task. Further, due to the lack of follow-up with a thorough clinical examination in general and in particular with more advanced diagnostic methods (such as MRI, OCT, and VEP), an underestimation of long-term health sequelae is likely [29, 30].

The poor outcome in methanol poisonings is primarily associated with the late diagnosis and delayed initiation of treatment. However, the rate of elimination of the toxic formate and correction of acidemia can theoretically play an important role in survival without long-term visual and CNS sequelae: IHD is superior to CRRT as regards to the elimination rate of methanol and formate, as well as time to correction of acidemia [23–26]. In addition, if CRRT is the only treatment available, elimination increases with increased blood and dialysate flow rates [25]. The formate anion is neurotoxic and given the statistically significant difference of serum creatinine concentration on admission and presumably the degree of acute kidney injury and glomerular filtration rate between the two groups, the endogenous formate clearance could potentially be lower in CRRT group than the mean half-life of 2.6 h shown by Hovda et al. [36]. Also, since there are no direct toxic effect on the kidneys per se like that seen in ethylene glycol, increased creatinine is likely rather a sign of more compromised circulation due to a more severe state of poisoning.

In our previously published study, we analyzed the prevalence and predisposing factors of brain damage and hemorrhages in survivors of acute methanol poisoning [15, 37, 38]. This group included 34 patients treated with RRT from the present study. The coagulation parameters and systemic anticoagulation were similar between IHD and CRRT groups. In 15 patients, brain hemorrhages were detected and nine patients had non-hemorrhagic brain lesions. No association between brain hemorrhages and systemic anticoagulation during dialysis was found: Brain hemorrhages might occur in severely poisoned patients treated without systemic anticoagulation, whereas treatment with high doses of heparin might not lead to brain hemorrhages [15].

The patients in the CRRT group were significantly more severely poisoned as regards to their consciousness, the degree of metabolic acidosis, the need for ventilator support and vasopressors/inotropes as compared to the IHD group. There were no differences in age, circumstances of poisoning, time to presentation, or start of hemodialysis. Thus, despite the fact that there were significantly more patients surviving without sequelae in the IHD group, the significance disappeared when the degree of acidemia and severity of poisoning was accounted for. This may imply two possible explanations: (1) The severity of the metabolic acidosis and the state of consciousness on admission are so important prognostic parameter of poor outcome that all other variables including the modality of enhanced elimination will remain second-rate to this or (2) the actual removal of the toxic metabolite and correcting of acidemia *per se* is more important than the time needed to correction.

Comparison of clinical effectiveness of two treatment modalities would be incomplete without the analysis of costs. In our previous study, the hospital costs in the patients treated with IHD were 5400 (IQR 1520–6910) versus 12,410 (IQR 5380–16,960) euros in the patients with CRRT. Therefore, IHD group had the trend to the lower total hospital costs. The difference between the total hospital costs of treatment with two modalities of RRT was on the border of significance (OR 0.70; 0.60–0.99 95% CI; *p* = 0.047) when adjusted for the severity of poisoning [39].

The next important issue is the possibility of hospital costs reduction by application of RRT in less severely poisoned patients (e.g., by shortening the duration of hospitalization). In the same study, we have found that the median total hospital costs in the patients treated without RRT were 1450 (IQR 650–2020) euros only, and after adjustment for the severity of poisoning, the total hospital costs were significantly lower in the patients treated without RRT than in the patients treated with any modality of RRT (OR 2.00; 1.40–3.00 95% CI; *p* < 0.001 for CRRT vs. no RRT; and OR 1.50; 1.10–2.10 95% CI; *p* = 0.015 for IHD vs. no RRT). Therefore, no cost reduction but rather an increase in total hospital costs was found if RRT modalities were applied in less severely poisoned patients [39].

Hemodynamic status is the most important decision factor for which RRT modality to use and the availability of RRT modalities during mass poisoning outbreak are the second determinant. Our data suggest that if a patient is hemodynamically unstable, CRRT is clearly indicated, thus no rationale for applying IHD “regardless of costs” and despite serious acute risks. If a patient is hemodynamically stable and both CRRT and IHD equipment is available in the medical facility, IHD would be the modality of choice from a kinetics point of view (elimination and acidemia correction rates), as well as hospital costs. Finally, if a patient is hemodynamically stable and IHD equipment is not available (e.g., in smaller medical facilities or due to high number of admitted patients), there is no rationale in transferring the patient to a larger medical facility with IHD equipment (due to the risk of deterioration during transportation and delayed hospital treatment) if CRRT is available without delay.

This study has several limitations, the most important one being lack of randomization. The study was not designed as a randomized trial, because the choice of the method of enhanced elimination in each case was conditioned by different factors, including the availability of dialyzing equipment in the different local hospitals, giving the possibility of inherent bias.

The numbers of the patients in both groups were relatively small, and most of the patients in both groups were severely poisoned “late presenters” (patients admitted to hospital later than 12 h after stop of toxic spirit ingestion). Twenty survivors were lost to follow-up and missing data were imputed using the last observation before discharge. The assumption that visual and neurological sequelae do not change in these patients during follow-up may not be appropriate; therefore, missing follow-up data present another limitation to the study. Further, the study was not controlled with regard to the treatment modalities. However, the present groups of patients treated with different modes of enhanced elimination represent the largest number of patients described in the literature of methanol poisonings treated with dialysis. They did not differ in age, time to presentation and diagnosis, most laboratory data, clinical signs on admission, or treatment provided.

## Conclusions

Apparently, more patients in our study seemed to survive without sequelae and less patients died when intermittent hemodialysis was being used as compared to continuous modalities. However, no differences in outcome were found when correcting for the severity of the poisoning (as primarily defined by the degree of metabolic acidosis). This can be attributed to the relative importance of the acidemia on prognosis. Even if removal of the toxic metabolites and correction of acidemia as soon as possible appear important, using whatever mode of dialysis available seems adequate, and the mode of dialysis should otherwise be chosen based on the circulatory status of the patient.

## Abbreviations

AACT/EAPCCT: American Association of Clinical Toxicology/European Association of Poison Centers and Clinical Toxicologists
AG: Anion gap
CNS: Central nervous system
CRRT: Continuous renal replacement therapy
CS: Central nervous system sequelae
CT: Computer tomography
ECTR: Extracorporeal treatment
EDD: Extended daily dialysis
GCS: Glasgow Coma Scale
ICU LOS: Intensive care unit length of stay
IHD: Intermittent hemodialysis
MRI: Magnetic resonance imaging
NAD: Nicotinamide adenine dinucleotide
OCT: Optical coherence tomography
OG: Osmolal gap
RCT: Randomized clinical trials
TIC: Toxicological Information Center
VEP: Visual evoked potentials
VS: Visual sequelae
